# Personalized prediction of disease activity in patients with rheumatoid arthritis using an adaptive deep neural network

**DOI:** 10.1371/journal.pone.0252289

**Published:** 2021-06-29

**Authors:** Maria Kalweit, Ulrich A. Walker, Axel Finckh, Rüdiger Müller, Gabriel Kalweit, Almut Scherer, Joschka Boedecker, Thomas Hügle

**Affiliations:** 1 Department of Computer Science, University of Freiburg, Freiburg Im Breisgau, Germany; 2 Department of Rheumatology, University Hospital Basel, Basel, Switzerland; 3 Division of Rheumatology, University Hospital Geneva, Geneva, Switzerland; 4 Department of Rheumatology, Kantonsspital Aarau, Switzerland; 5 SCQM Foundation, Zürich, Switzerland; 6 Department of Rheumatology, Lausanne University Hospital (CHUV) and University of Lausanne, Lausanne, Switzerland; Lingnan University, HONG KONG

## Abstract

**Background:**

Deep neural networks learn from former experiences on a large scale and can be used to predict future disease activity as potential clinical decision support. AdaptiveNet is a novel adaptive recurrent neural network optimized to deal with heterogeneous and missing clinical data.

**Objective:**

We investigate AdaptiveNet for the prediction of individual disease activity in patients from a rheumatoid arthritis (RA) registry.

**Methods:**

Demographic and disease characteristics from over 9500 patients and 65.000 visits from the Swiss Quality Management (SCQM) database were used to train and evaluate the network. Patient characteristics, clinical and patient reported outcomes, laboratory values and medication were used as input features. DAS28-BSR served as a target to predict active RA and future numeric individual disease activity by classification and regression.

**Results:**

AdaptiveNet predicted active disease defined as DAS28-BSR >2.6 at the next visit with an overall accuracy of 75.6% (SD +- 0.7%) and a sensitivity and specificity of 84.2% (SD +- 1.6%) and 61.5% (SD +- 3.6%), respectively. Prediction performance was significantly higher in patients with a disease duration >3 years and positive rheumatoid factor. Regression allowed forecasting individual DAS28-BSR values with a mean squared error (MSE) of 0.9 (SD +- 0.05). This corresponds to a 8% deviation between estimated and real DAS28-BSR values. Compared to linear regression, random forest and support vector machines, AdaptiveNet showed an increased performance of over 7% in MSE. Medication played a minor role in the prediction of RA disease activity.

**Conclusion:**

AdaptiveNet has a superior capacity to predict numeric RA disease activity compared to classical machine learning architectures. All investigated models had limitations in low specificity.

## Introduction

Rheumatoid arthritis (RA) is a chronic inflammatory disorder in which disease activity fluctuates over time. The advent of targeted synthetic and biologic medication, along with early and treat-to-target strategies have substantially improved patient care. However, sustained remission still is only achieved in around 30% indicating room for improvement either by new drugs or alternative treatment strategies [1]. EULAR/ACR recommendations suggest treatment modification after three to six months if the set target is not reached, regardless of the presence or absence of individual risk factors for poor outcome [2]. Given the increasing number of available drug combinations, the delay in finding the best individual treatment can be substantial. The practical role of biomarkers to predict individual chances of good therapeutic response remains limited [3,4]. Classical predictors such as female gender or rheumatoid factor positivity but also more complex prediction models have been shown to be unreliable to forecast individual response to methotrexate after 3–6 months [5]. There are also no clear recommendations on treatment de-escalation in case of stable disease despite disease activity-guided dose optimization of biologic being efficient and cost-effective [6,7]. In other words, over- or undertreatment in RA is common, potentially resulting either in destructive disease flares or unnecessary side effects and costs [8].

Machine learning (ML) is increasingly used for disease detection, stratification and prediction both in at risk populations and established disease in various fields of medicine, including rheumatology [9,10]. Among conventional ML methods, random forests have shown a higher accuracy to predict disease activity compared to support vector machines (SVM) or logistic regression in non-rheumatic disorders such as heart failure or diabetes [11]. Fuzzy cognitive maps is another increasingly used ML-method for clinical prediction tasks and decision support [12]. Using data from electronic medical records (EMR), ML has successfully predicted RA flares in a small number of RA patients by a random forest as a classical ML method [13,14]. Only few data exist on deep learning (DL) in rheumatology. DL is a specialized subfield within ML which relies on neural networks and offers a higher productivity and flexibility compared to conventional ML techniques [15]. Norgeot et al. applied DL to EMR data in 820 RA patients for the prediction of disease activity by classification [16]. To predict the category of low disease activity, a remarkable AUC score of 0.91 was achieved in a test set of 116 patients. This study was limited by low patient numbers and lack of complete data on medication. Using the Swiss Quality Management (SCQM) database [17] for rheumatic diseases, we recently described a novel adaptive deep neural network (AdaptiveNet), per se showing superior results compared to a naive rule-based baseline, a random forest and a conventional fully-connected deep neural network architecture in the prediction of disease activity in RA patients [18]. AdaptiveNet projects patient data of events such as visits or medication adjustments to the same latent space using multiple encoder networks. The sorted list of encoded events is pooled by a long short-term memory (LSTM) to account for temporal dependencies and generates a fixed-length encoded patient history [19]. The main advantage of this architecture is better handling of heterogeneous and missing clinical data.

The study presented here aims to characterize this deep neural network to forecast individual disease activity both categorically and numerically as a potential tool for clinical decision support.

## Methods

### Study design and data source

The dataset used is the Swiss Clinical Quality Management in Rheumatic Disease (SCQM) registry, a national multicenter database containing longitudinal data from clinically diagnosed RA patients. The registry was established in 1997 to prospectively follow RA patients [17]. RA diagnoses are made clinically by board-certified rheumatologists. Follow-up for the registry involves one to four annual visits with physical examination, (yearly) hand radiographs, disease activity scores (e.g., DAS28), laboratory tests (e.g., erythrocyte sedimentation rate [esr]) and several patient self-report questionnaires (e.g., SF [short form] 12). Clinical information is also usually updated every time a patient changes antirheumatic therapy. Clinical characteristics of the patients included in this study are seen in Table 1. The study was approved by the regional ethics committee “Commission cantonale Vaud d’éthique de la recherche sur l’être humain” (ID 2020–000333). All individuals willing to participate sign an informed consent form before enrolment, in accordance with the Declaration of Helsinki.

**Table 1:** Clinical characteristics and input features.

Numerical	Missing [%]	Mean (+- SD)
Age*	0	58.8 (+- 13.0)
Minimal Disease Activity*	1.6	1.3 (+- 1.1)
Disease Duration*	2.7	12.2 (+- 9.5)
Number swollen joints**	6.6	3.3 (±4.6)
Number painful joints**	6.9	3.5 (±5.3)
BSR**	14.6	18.5 (±17.1)
DAS28-BSR**	16.4	3.2 (±1.4)
Pain level (0–10)**	22.4	3.3 (±2.7)
Disease activity index (RADAI)**	22.7	3.4 (±2.7)
HAQ score**	27.8	0.8 (±0.7)
Weight [kg]**	36.5	70.7 (±15.6)
Height [cm]**	40.8	165.3 (±12.2)
**Categorical**		**Values [%]**
Female gender*	0	74
Rheumatoid factor positive*	9.1	62.9
Rheumatoid factor negative*	9.1	28
Anti-CCP*	31.6	positive [42.4]
		negative [26.0]
Morning stiffness (RADAI)**	22.7	None [36.8]
		All day [1.9]
		<0.5 hour [15.4]
		0.5–1 hour [12.0]
		>4 hours [1.6]
		12 hours [6.1]
		24 hours [3.5]
Smoker**	60.2	Never [18.2]
		Current [9.3]
		Former [12.3]
Treatment***	-	Methotrexate [24.1]
		Prednisone [16.8]
		Adalimumab [7.9]
		Etanercept [7.3]
		Tocilizumab [4.0]
		Abatacept [4.0]
		Rituximab [3.5]
		Golimumab [2.4]
		Other [30.1]
Treatment type***	-	Prednisone [18.8]
		DMARD [24.1]
		Biologic [29.0]
		Other [30.1]
Prednisone dose***	-	None [41.3]
		< 10mg [9.6]
		10-15mg [12.6]
		>15mg [36.5]

The corresponding number of missing values, mean and standard deviation (SD) for numerical variables, and the distribution in percent for categorical variables for 28601 visits. All listed characteristics are used as input features for AdaptiveNet as either general patient features*, visit features** or medication features***. BSR: blood sedimentation rate, anti-CCP: anti citrullinated peptide antibodies. HAQ: health assessment questionnaire. DMARD: disease modifying anti-rheumatic drug.

### Prediction target and input features

To predict disease activity, we used the RA activity score DAS28-BSR at next visit as target variable. DAS28-BSR stands for disease activity score and assesses 28 joints for tenderness and swelling as well as subjective disease activity of the patient and blood sedimentation rate (BSR) as laboratory marker for inflammation [20]. We only considered visits with complete DAS28-BSR scores. We used age, gender, weight, disease duration, BSR, CRP (C-reactive protein), swollen joint count, painful joint count, rheumatoid factor, anti-CCP (cyclic citrullinated peptide), treatment, smoking status, HAQ (health assessment questionnaire), morning stiffness, EuroQol (as instrument for measuring the generic health status), disease activity and pain level as potential predictors (Table 1). For antirheumatic therapy, we used the individual drugs, as well as broader drug categories of biologic (b) or conventional (cs) disease modifying anti-rheumatic drugs (DMARD) and prednisone dose strata, respectively. Duration of therapy since adjustment was also assessed. For training and evaluation of the predicted target variable we considered follow-up visits between 1 month and 1 year. All visits and medication data of the last 5 years were considered.

### Classification and regression

For classification, we defined two disease states, active disease (DAS28-BSR > 2.6) and remission (DAS28-BSR ≤ 2.6) at next visit [21]. Prediction performance was measured by accuracy, sensitivity, specificity and area under the curve (AUC) score. For visualization, we used the Receiver Operating Characteristic Curve (ROC), which shows the tradeoff between sensitivity (true positive rate) and specificity (1—false positive rate). For statistical difference, we compared the area under the ROC with a Welch’s t-test and considered p≤0.05 as significant. In order to predict numeric values of the target variable (DAS28-BSR), we applied a regression model and predicted the expected change of DAS28-BSR to the subsequent visit. Performance was measured by MSE as an estimator of the deviation between the estimated and actual values. To evaluate the models, we split the dataset into a training set and 5 different test sets by using 5-fold cross-validation. The test set contains 20% of the data, the training set 80% of the data.

### Data processing procedure and modelling

**Fig 1:**
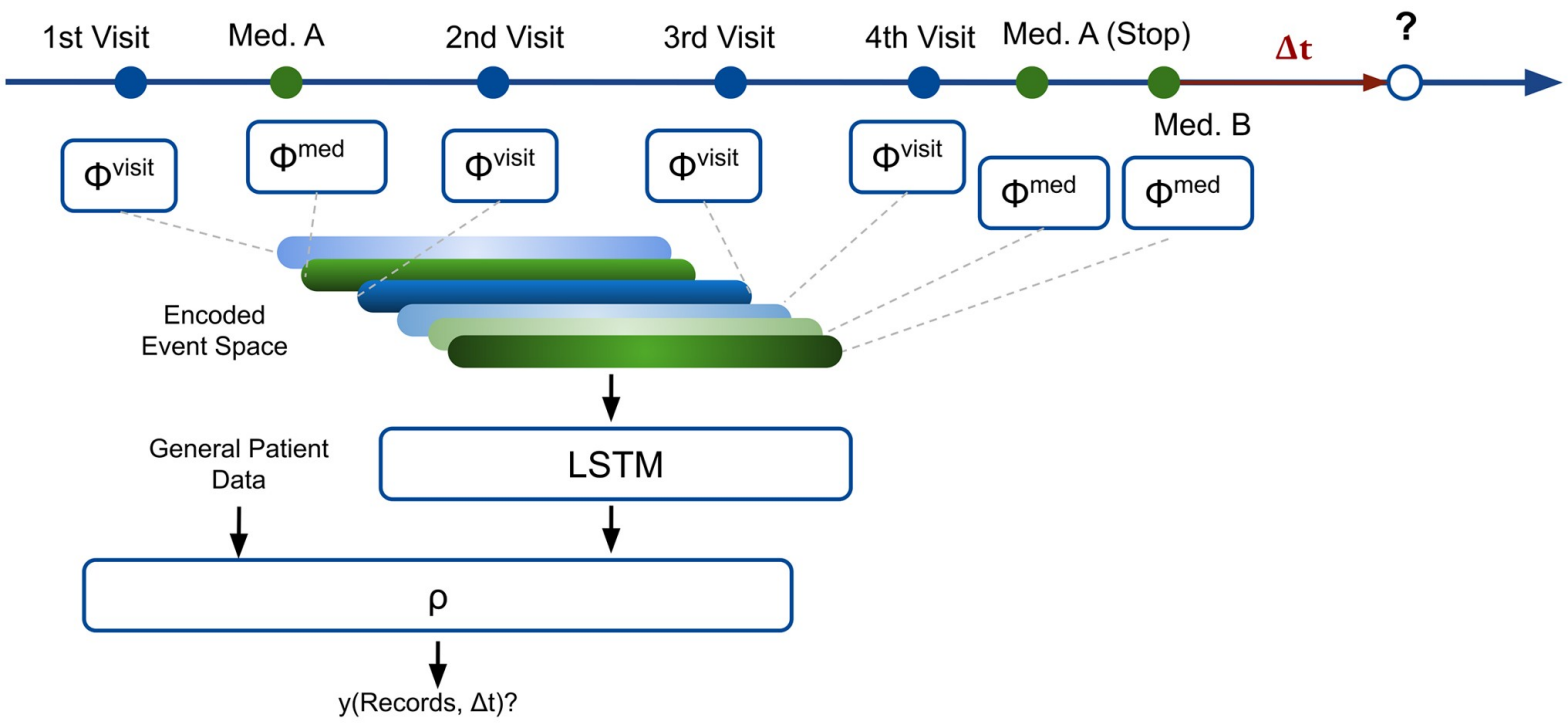
Deep neural network architecture (AdaptiveNet). All visits and medication adjustments are projected to latent vectors of the same size using encoder networks ϕ^visits^ and ϕ^meds^. Latent vectors are sorted according to dates and fed into a Long Short-term Memory (LSTM) to create a latent vector describing the full patient history. The final prediction is computed by the network module ρ, exploiting the patient history with general patient information.

Classification and regression was performed with AdaptiveNet, a dynamic and recurrent deep neural network architecture, designed for chronological clinical data [18]. In short, AdaptiveNet encodes all former clinical events of a patient (here: visits and medication adjustments) to the same latent space using multiple fully-connected encoder networks in order to align the corresponding output vectors (Fig 1). Sorted lists of these encoded clinical events are pooled by an LSTM to compute a fixed-length encoding, representing the 5-year patient history and accounting for temporal dependencies. The final output is computed by a fully-connected network module, using the encoded patient history and additional features containing general time-independent patient information as input. For preprocessing, all features were scaled in the range [0, 1]. The architecture of AdaptiveNet is shown in the S1 Table. For regression and classification, the Adam optimizer with a learning rate of 10^−4^ was used [22]. Batch size was set to 256. We used loss of MSE for regression and binary cross-entropy for classification.

As baselines, we used a random forest with a maximum depth of 12 and 100 estimates, linear and logistic regression models and an SVM with regularization parameter C = 10 for regression and radial basis function as kernel type. Hyperparameters were tuned for all approaches, including the baselines, using random search in the configuration space shown in S2 Table. For feature importance, the influence on the mean decrease in weighted impurity was calculated for each feature using a Random Forest.

## Results

### Categorical prediction of disease activity by classification

In total, 28.601 visits with corresponding disease activities were extracted. Over a maximal observed history length of 5 years, patients had 6.3 (±5.3) visits and 2.5 (±2.7) medication adjustments. For the classification task DAS28-BSR>2.6 at next visit (mean interval 8.1 +-2.9 months from initial visit), AdaptiveNet had an accuracy of 75.6% (SD +- 0.7%) and an AUC score of 0.728 (SD +- 0.01) (Table 2). A random forest showed an accuracy of 75.0% (SD +- 0.93%) and an AUC of 0.71 (SD +- 0.01). Using logistic regression, we achieved an accuracy of 73.5% (SD +- 1.45%) and an AUC of 0.70 (SD +- 0.012). The SVM showed 73.3% accuracy (SD +- 0.97%) and an AUC of 0.69 (SD +- 0.011).

**Table 2:** Performance of an AdaptiveNet model for prediction of active disease in test sets containing different patient subsets.

	No. of visits	Accuracy	Sensitivity	Specificity	AUC	MSE
All patients (Max. observation time 5 years)	28601	0.75 (+- 0.007)	0.84 (+- 0.01)	0.61 (+- 0.03)	0.72 (+- 0.01)	0.90 (+- 0.05)
Age ≥ 50 years	21653	0.76 (+- 0.01)	0.85 (+- 0.01)	0.59 (+- 0.04)	0.72 (+- 0.01)	0.87 (+- 0.06)
Age < 50 years	6948	0.74 (+- 0.01)	0.80 (+- 0.02)	0.66 (+—0.04)	0.73 (+- 0.01)	0.98 (+- 0.06)
Disease duration < 3 years	3257	0.70 (+- 0.01)	0.83 (+- 0.04)	0.56 (+- 0.07)	0.70 (+- 0.02)	1.05 (+- 0.13)
Disease duration ≥ 3 years	24558	0.75 (+- 0.008)	0.83 (+- 0.01)	0.62 (+- 0.03)	0.73 (+- 0.01)	0.88 (+- 0.05)
Rheumatoid factor positive	21501	0.76 (+- 0.007)	0.85 (+- 0.01)	0.60 (+- 0.04)	0.73 (+- 0.01)	0.91 (+- 0.05)
Rheumatoid factor negative	6607	0.71 (+- 0.02)	0.77 (+- 0.06)	0.64 (+- 0.03)	0.70 (+- 0.01)	0.87 (+- 0.08)
Anti-CCP negative	6228	0.74 (+- 0.01)	0.82 (+- 0.004)	0.626 (+- 0.03)	0.72 (+- 0.01)	0.92 (+- 0.05)
Anti-CCP positive	13084	0.74 (+- 0.02)	0.78 (+- 0.06)	0.67 (+- 0.02)	0.73 (+- 0.02)	0.85 (+- 0.07)
Male	7174	0.75 (+- 0.02)	0.779 (+- 0.03)	0.72 (+- 0.05)	0.75 (+- 0.01)	0.97 (+- 0.07)
Female	21427	0.75 (+- 0.01)	0.85 (+- 0.01)	0.56 (+- 0.02)	0.71 (+- 0.007)	0.88 (+- 0.07)

Accuracy, sensitivity, specificity, and area under the curve (AUC) indicate performance for classification, Mean Squared Error (MSE) for regression. All results show mean and standard deviation over 5 different cross-validation folds. Anti- CCP: Citrullinated peptide antibodies.

**Fig 2:**
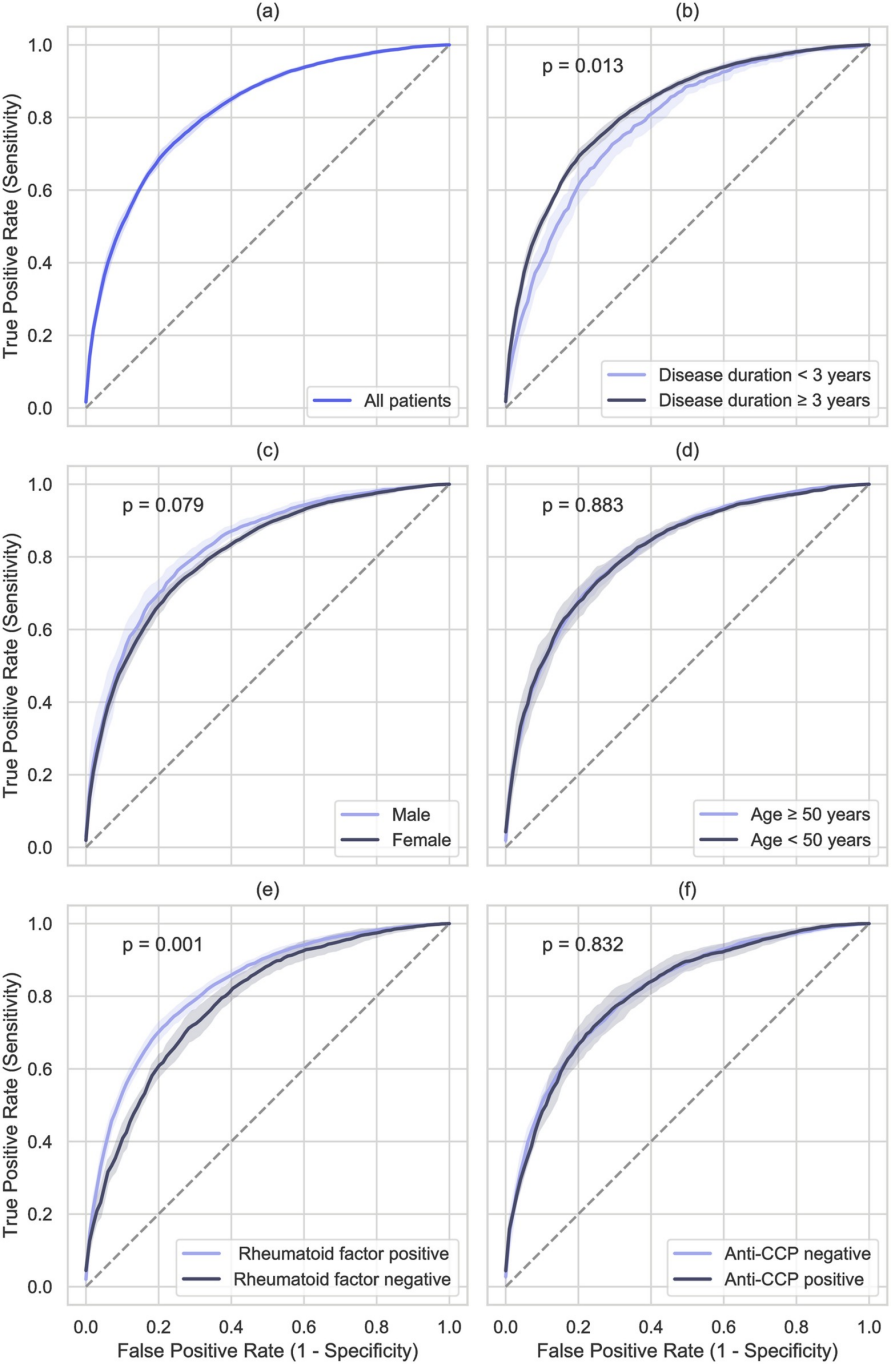
Classification performance of AdaptiveNet to predict active disease (DAS28-BSR>2.6) in different patient subsets shown by Receiver Operating Characteristic Curves. Accuracy and corresponding AUCs are indicated in Table 1.

The Receiver Operating Characteristic Curve (ROC) for AdaptiveNet is shown for all patients (Fig 2a) and for different clinical variables (Fig 2b-2f). The performance was significantly higher in patients with longer disease duration (p = 0.013) and positive rheumatoid factor (p = 0.001). Male gender showed a positive trend for a better performance (p = 0.079). Data from patients aged >50 years (Fig 2c) and from anti-CCP positive patients (Fig 2f) achieved a higher specificity but no significantly increased performance compared to patients aged <50 years or anti-CCP negative patients.

### Numerical prediction of disease activity by regression

**Fig 3:**
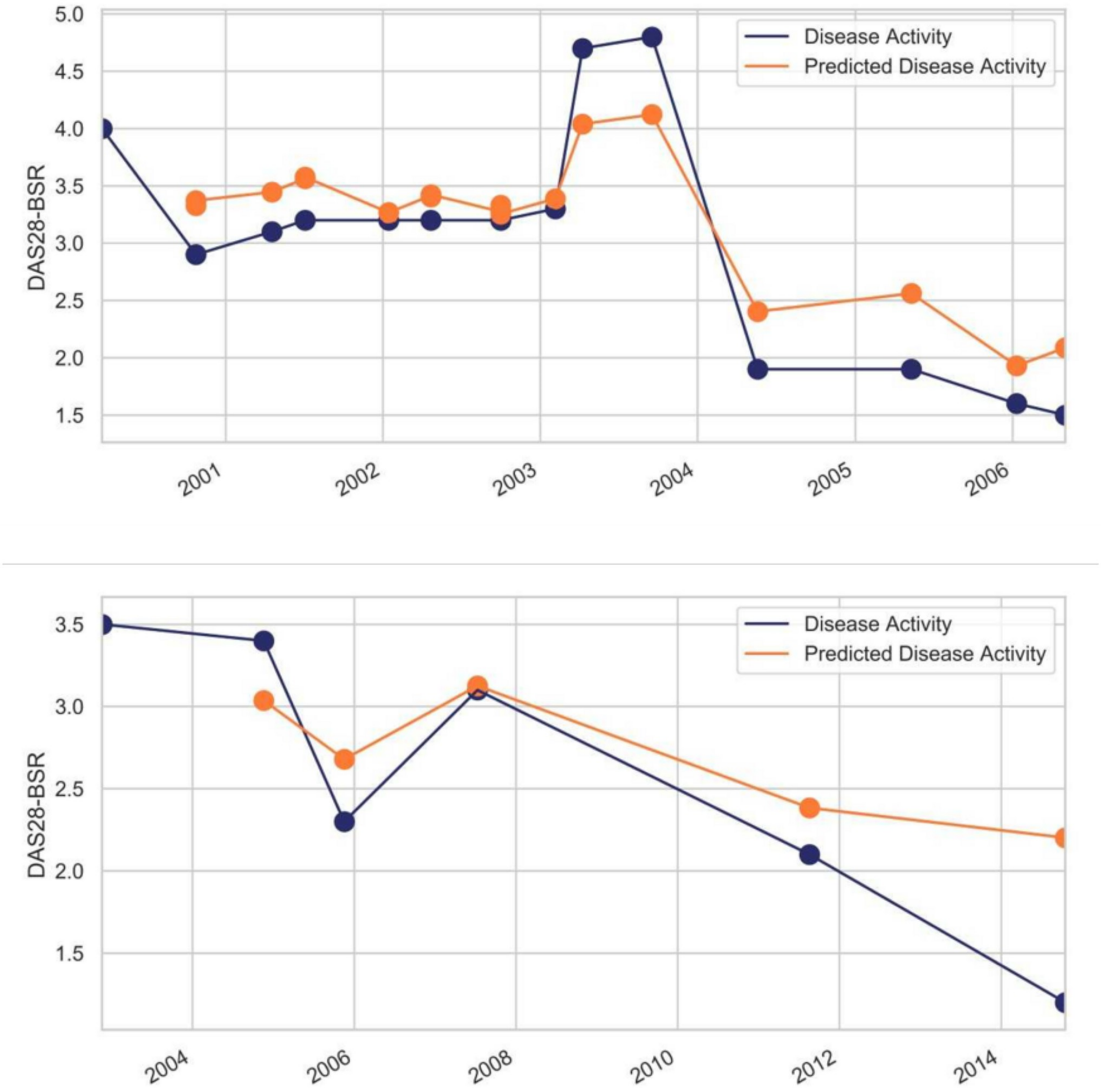
Examples of true disease activity and corresponding predictions of AdaptiveNet by regression analysis. Predictions are made step to step from the current to next visit.

AdaptiveNet was applied to predict the numerical DAS28-BSR value at the next visit by regression on an individual level. When trained on data from all patients, we obtained an overall MSE of 0.90 (SD +- 0.05), which corresponds to a 8% deviation between estimated and real DAS28-BSR values (Table 2). Fig 3 shows exemplary results for two patients with individual forecasts of DAS28-BSR values over time. A general capacity of the model to predict disease flares as well as response to treatment could be demonstrated. Predicted DAS28-BSR amplitudes during flares were lower than real values and smaller variations of disease activity were not predictable. We obtained better results for patients with disease duration >3 years, age >50 and positive anti-CCP antibodies (Table 2). In contrast to classification, regression had lower MSE values and thus performed better in female and RF-negative patients. The linear regression model showed a lower performance with a significantly higher MSE compared to AdaptiveNet (0.97 SD +- 0.06). MSE of the random forest was 0.963 (SD +- 0.05) and 0.978 (SD +- 0.06) for the SVM. The advantage of AdaptiveNet over a fully-connected neural network has been shown previously [18].

### Feature importance

Feature importance was determined by a random forest to define the relative importance of variables for disease prediction (S1 Fig). Apart from the target variable itself, the number of painful joints, longer disease duration and age turned out to be the most relevant factors, followed by medication in general, time point of last medication adjustment, number of swollen joints, and HAQ. The importance of medication type (csDMARD vs. bDMARD or corticosteroids) for the prediction of DAS28-BSR was only marginal. Infliximab, tocilizumab and steroids had a slightly higher influence than csDMARDs or other bDMARDs in predicting disease activity.

## Discussion

This study demonstrates a comprehensive classification and regression analysis using a novel deep learning architecture on a RA dataset. Our algorithm allowed individual predictions of DAS28-BSR values at next visit with an acceptable deviation of 8% compared to real values. We postulate that concrete numerical predictions of disease activity, rather than mere classification into high or low risk patients might facilitate the application of DL predictions in clinical practice e.g. to optimize treat-to-(predicted)-target strategies or setting control intervals.

AdaptiveNet outperformed linear and logistic regression, a random forest and a support vector machine as basic ML methods. This confirms the problem of incomplete and timely inhomogeneous data for ML from registries or from electronic medical records [23]. Improvement is required concerning the relatively low specificity, the main limitation of all investigated ML methods in this analysis. Potentially, the combination with other ML methods such as using pre-trained generative models or K-nearest neighbor (KNN) methods could further improve performance of AdpativeNet [24,25]. To further improve the performance, larger datasets through -omics or digital biomarkers e.g. by wearables and patient reported outcomes could be taken into account. For example, ML algorithms using data from activity tracker have been described to monitor disease activity in RA and to detect flares as inexpensive data sources with minimal patient burden [26].

We investigated the influence of different clinical variables on the prediction performance in RA. As a further new finding, long disease duration and rheumatoid factor positivity increase the predictability of the active disease by classification. This information could be of importance e.g. for patient selection in future ML-assisted clinical trials. In contrast to classification, the prediction of numeric DAS28-BSR by regression performed better in females and in anti-CCP positive patients. The reason for the different role of rheumatoid factor and anti-CCP status in classification versus regression analysis remains to be investigated. Classification tasks are prone to overfitting to the old class, e.g. predicting no change to the previous situation. Patients in remission for a long period likely will stay in remission, or vice versa, patients resistant to multi-line treatment will more likely remain in active disease. Female, anti-CCP positive patients per se having a higher risk of clinical progression might be less sensitive for overfitting and thus more suitable for regression analysis. Prediction performance in regards of treatment history has not been performed in this study. To some extent surprising, medication was less important for the prediction of disease activity than age or disease duration. The reason for this might be explained by limited effectiveness after multi-line treatments or vulnerability of DAS28-BSR as target variable to confounding factors as e.g. fibromyalgia. The slightly higher performance of infliximab to forecast disease activity is reasonable from a clinical perspective by intravenous application and higher doses. Whether DL is able to predict drug survival or individual treatment responses needs to be evaluated.

As a limitation of this study we did not compare AdaptiveNet to statistical prediction models not based on ML. On the other hand, the weakness of classical prediction models e.g. for response to methotrexate has been pointed out in a recent meta-analysis, indicating the need for novel prediction models [5]. Potentially, disease features such as epigenetics but also lifestyle, sleep or nutrition contribute to prediction performance more than expected, notably when pain is part of the predicted target such as in the DAS28 score. Thus, further studies need to investigate the performance of DL using alternative input and target features including other markers for disease activity than DAS28-BSR.

Taken together, AdaptiveNet is superior to conventional ML methods in predicting disease activity in RA patients. We also provide evidence which clinical features increase predictability of this model. We are convinced that DL will play an increasing role to improve patient care and to foster personalized treatment and shared-decision making in patients with RA. Numeric forecast of disease activity may open the way for a ´treat-to-predicted-target´ stewardship which could be more time-efficient than conventional treat-to-target approaches. Prospective trials will be necessary to prove efficacy, safety and cost effectiveness of ML-assisted care in arthritis.

## Supporting information

S1 Fig Feature importance. The relative importance of variables for prediction of active disease is computed by a Random Forest, considering features of the last visit and last medication. Drug classes and individual drugs are indicated separately in the lower part.
(DOCX)

S1 Table Architecture of AdaptiveNet. FC denotes fully-connected layers, seq^(·)^ is the length of the variable-sized lists of visit and medication events, B the batch size, 21 the number of visit features and 18 the number of medication features, respectively. (*) The weights of the second fully-connected layers are shared between the two encoders.
(DOCX)

S2 Table Hyperparameter optimization. Configuration spaces for all approaches. The best performing parameter setting is shown in bold.
(DOCX)

S1 Rawdata (CSV)
