# Supplementary material for: Personalized prediction of disease activity in patients with rheumatoid arthritis using an adaptive deep neural network
Source: PLoS One. 2021 Jun 29;16(6):e0252289. doi: 10.1371/journal.pone.0252289 (PMC8241074; doi:10.1371/journal.pone.0252289)

**Supplement 3: Feature importance.** The relative importance of variables for prediction of active disease is computed by a random forest, considering features of the last visit and last medication. Drug classes and individual drugs are indicated separately in the lower part.


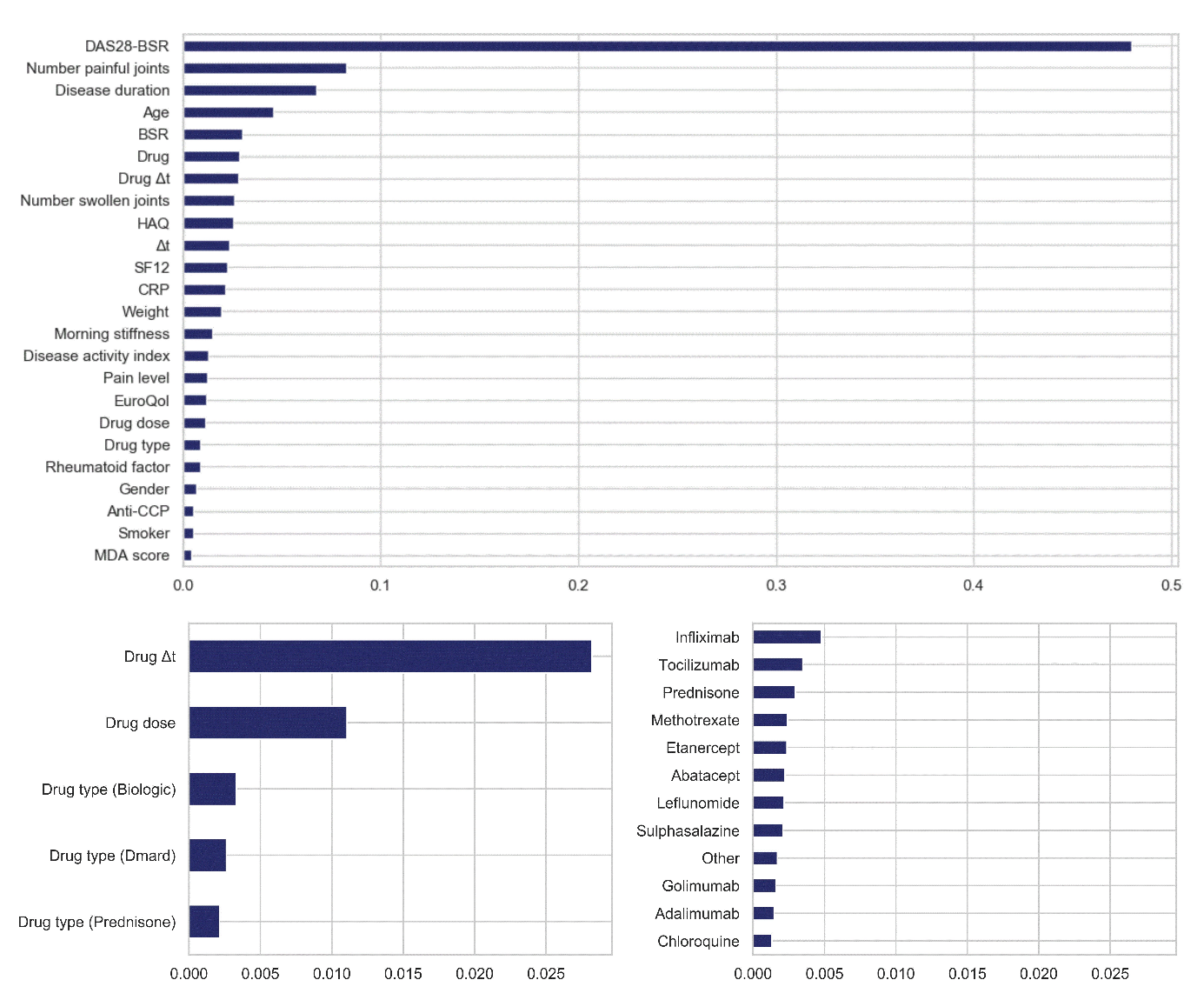

Supplement: S1 Fig — The relative importance of variables for prediction of active disease is computed by a Random Forest, considering features of the last visit and last medication. Drug classes and individual drugs are indicated separately in the lower part. (DOCX) [file pone.0252289.s001.docx]
