# Supplementary material for: Personalized prediction of disease activity in patients with rheumatoid arthritis using an adaptive deep neural network
Source: PLoS One. 2021 Jun 29;16(6):e0252289. doi: 10.1371/journal.pone.0252289 (PMC8241074; doi:10.1371/journal.pone.0252289)
Supplement: S1 Table — FC denotes fully-connected layers, seq(·) is the length of the variable-sized lists of visit and medication events, B the batch size, 21 the number of visit features and 18 the number of medication features, respectively. (*) The weights of the second fully-connected layers are shared between the two encoders. (DOCX) [file pone.0252289.s002.docx]

Supplementary data

**Supplement 1**: Architecture of AdaptiveNet. FC denotes fully-connected layers, seq^(·)^ is the length of the variable-sized lists of visit and medication events, B the batch size, 21 the number of visit features and 18 the number of medication features, respectively. (*) The weights of the second fully-connected layers are shared between the two encoders.

| **AdaptiveNet** |
| --- |
| Input(B x seq^visit^ x 21) and Input(B x seq^med^ x 18) |
| ɸ^visit^: FC(100), FC(100)*, ɸ^med^: FC(100), FC(100)*  LSTM(·) |
| concat(·, Input(B x 8)) ⍴: FC(100), FC(100) |
| Regression: Linear(1)     Classification: Linear(2) |
