# Supplementary material for: Personalized prediction of disease activity in patients with rheumatoid arthritis using an adaptive deep neural network
Source: PLoS One. 2021 Jun 29;16(6):e0252289. doi: 10.1371/journal.pone.0252289 (PMC8241074; doi:10.1371/journal.pone.0252289)
Supplement: S2 Table — Configuration spaces for all approaches. The best performing parameter setting is shown in bold. (DOCX) [file pone.0252289.s003.docx]

**Supplement 2:** Hyperparameter Optimization. Configuration spaces for all approaches. The best performing parameter setting is shown in bold.

| **Model** | **Parameter** | **Configuration Space** |
| --- | --- | --- |
| AdaptiveNet | ɸ^(·)^: hidden dimension  ɸ^(·)^: num hidden layers  ⍴: hidden dimension  ⍴: num hidden layers  dropout rate | [32, 64, **100**]  [**2**, 3]  [64, **100**, 200]  [1,**2**,3]  [**0.0**, 0.1, 0.25] |
| Random Forest | Max depth | [8, 10, **12,** 15] |
| Linear Regression | - | - |
| Support Vector Machine (SVM) | Regularization parameter | [0.1, 1, **10**] |
